# Microbial influence on blood pressure: unraveling the complex relationship for health insights

**DOI:** 10.20517/mrr.2023.73

**Published:** 2024-03-18

**Authors:** Alexander Machado Cardoso

**Affiliations:** Department of Biology, Rio de Janeiro State University, Rio de Janeiro 23070200, Brazil.

**Keywords:** Cardiovascular risk, gut microbiota, hypertension, lactobacillus, machine learning, microbiome-host interactions

## Abstract

Hypertension, a critical global health concern, is characterized by persistent high blood pressure and is a major cause of cardiovascular events. This perspective explores the multifaceted implications of hypertension, its association with cardiovascular diseases, and the emerging role of the gut microbiota. The gut microbiota, a dynamic community in the gastrointestinal tract, plays a pivotal role in hypertension by influencing blood pressure through the generation of antioxidant, anti-inflammatory, and short-chain fatty acids metabolites, and the conversion of nitrates into nitric oxide. Antihypertensive medications interact with the gut microbiota, impacting drug pharmacokinetics and efficacy. Prebiotics and probiotics present promising avenues for hypertension management, with prebiotics modulating blood pressure through lipid and cholesterol modulation, and probiotics exhibiting a general beneficial effect. Personalized choices based on individual factors are crucial for optimizing prebiotic and probiotic interventions. In conclusion, the gut microbiota’s intricate influence on blood pressure regulation offers innovative perspectives in hypertension therapeutics, with targeted strategies proving valuable for holistic blood pressure management and health promotion.

## INTRODUCTION

Hypertension is a multifactorial disease with genetic and environmental factors contributing to its development. The pathophysiology of hypertension is complex and involves a variety of physiological mechanisms, including salt intake, obesity and insulin resistance, the renin-angiotensin system, and the sympathetic nervous system^[1]^. Other factors that have been evaluated include genetics, endothelial dysfunction, low birth weight and intrauterine nutrition, and neurovascular anomalies^[2]^. Environmental factors such as cold temperature, high altitude, loud noises, stress, and ambient air pollutants can also significantly increase arterial blood pressure (BP), and chronic exposures may be capable of promoting the development of sustained hypertension^[3]^.

Being a major risk factor for cardiovascular events and the primary cause of adult mortality, hypertension is a serious global public health concern that is characterized by persistently high BP. It has been acknowledged as the primary risk factor, or as one of the most powerful risk factors, for the development of a variety of diseases that are acquired over the course of a person’s lifetime. These include a range of illnesses such as heart disease, heart arrhythmias, valvular heart diseases, cerebral stroke, and kidney failure^[4]^. Due to the widespread impact of hypertension, multifaceted approaches to its prevention and treatment are imperative as it plays a critical role in the development of numerous diseases^[4,5]^.

Extensive cohort studies have conclusively demonstrated that high BP is a significant risk factor for chronic kidney disease, atrial fibrillation, and heart failure^[6]^. The most important modifiable risk factor associated with early cardiovascular disease, when considering population attributable risk, is hypertension. A thorough systematic review and meta-analysis showed a progressive and graded relationship between various BP categories and young adults’ risk of cardiovascular events. Interestingly, this risk showed a progressive increase over different BP increments. In the context of public health, these findings highlight the critical role that BP management plays in reducing the risk of cardiovascular morbidity and the significance of focused interventions^[3]^.

Recent studies have highlighted the revolutionary potential of high-throughput sequencing and integrative multi-omics approaches in deciphering the complex mechanisms underlying hypertension. Thanks to the power of bioinformatics and high-throughput technologies, an extensive global genome exploration has been made possible, revealing dysregulated genes that are closely associated with hypertension^[7]^. Beyond genomics, novel insights into the molecular basis of hypertension have been provided by postgenomic biomarkers derived from metagenomics, transcriptomics, proteomics, glycomics, and lipidomics^[8]^.

In order to comprehend the intricate interplay between internal and external risk variables that underpin the pathogenesis of hypertension, multi-omics analyses across the spectrum of continuous variations in BP values have to be incorporated^[9]^. These studies have the potential to identify risk factors, clarify the complex molecular landscape of hypertension, and track treatment outcomes. It is crucial to recognize that there are inherent computational and practical challenges with performing an integrative analysis of omics data. As of right now, this method is still not widely used in hypertension research^[10]^. The combination of high-throughput sequencing and multi-omics approaches is expected to make a substantial contribution to our understanding of hypertension at the molecular level as these challenges are rigorously addressed and methodologies progress. This development could lead to the implementation of precision medicine in the treatment of hypertension and more focused therapeutic approaches^[11]^.

The objective of this perspective is to provide insight into whether beneficial microbes, which are microorganisms that have positive effects on host health, could effectively contribute to enhancing the management of high BP. These microorganisms are typically found in the gastrointestinal tract, particularly in the colon, where they contribute to various physiological processes and help maintain homeostasis^[12]^. The microbiome refers to the diverse community of microorganisms that interact with their hosts in a range of relationships, including symbiotic, commensal, and pathogenic. Symbiosis, specifically, involves mutually beneficial exchanges between the host and microbiota, contributing to the maintenance of homeostasis and overall health^[13]^. The examination of microbial communities, their interactions, evolution, and the identification of novel molecular pathways in hypertension can be tackled through the application of computational predictive methods and functional genomics^[14]^. In an effort to understand the mechanistic relationships between the microbiome (the complete collection of genetic materials derived from all the microorganisms inhabiting a given environment) and the host, computational biology and machine learning techniques have been developed. The methodologies encompass the identification of novel molecular mechanisms and the profiling of heterogeneous datasets that document changes in the microbiome and host responses at the community level^[15]^.

These methods have been used to examine and deduce the interactions between host molecular components and the microbiota in a variety of disease contexts. Evidence of possible connections between the gut microbiota (GM) and BP in the setting of hypertension has been found, and the magnitude of these interactions has been studied^[16]^. Additionally, despite having similar GM profiles, a study that integrated microbiome pathway analysis and machine learning found differences in microbial gene pathways between participants with hypertension and those with normotension^[17,18]^. The downregulation of the nitrite transporter and salt transport system genes in hypertension individuals was especially noteworthy. Moreover, a significant increase in acetate-CoA ligase, an enzyme involved in the production of butyrate that can lower BP in experimental hypertension, was observed^[18]^. Consequently, functional genomics and computational predictive approaches can offer important new perspectives on the investigation into microbial communities and their connection to hypertension^[17]^.

In addition to its pivotal role in BP regulation, the GM exerts multifaceted effects on host health and disease, such as intestinal vascularization and gut barrier regulation^[19]^. These effects span diverse physiological processes, including immune homeostasis, physiological remodeling, and the maintenance of gut barrier integrity. The intricate interplay between the microbiota and the host extends beyond mere symbiosis, encompassing mechanisms that discriminate between homeostasis and dysbiosis. This continuous interaction exerts profound impacts on the host’s physiology and pathology. To provide a comprehensive overview of gut microbial effects on host health and disease, it is important to elucidate not only the specific relationship between the microbiota and BP regulation but also its broader implications for overall health.

## GUT MICROBIOTA CONTROLS BLOOD PRESSURE

The gastrointestinal tract is colonized by a complex and constantly evolving population of microorganisms known as the GM. It has co-evolved with the host and is composed of bacteria, archaea, and eukaryotes, including viruses that infect prokaryotes (bacteriophages) and eukaryotic viruses that infect human cells^[20]^. This dynamic community significantly influences host physiology during both normal physiological states (homeostasis) and pathological conditions (illness)^[21]^. For example, it has been demonstrated that the proportion of the bacterial species Firmicutes to Bacteroidetes affects both health and disease^[22]^. The microbiota in the gut plays an essential role in the immune system and metabolism, as well as in the fermentation of substrates that are not digestible, such as dietary fibers^[23]^. Several variables, including host genetics, environment, drugs, and diet, affect the GM’s composition. Studies have indicated that some chronic gastrointestinal diseases may be influenced by the GM, and that modifying it may be a useful therapeutic strategy to address these disorders^[24]^.

Additionally, it is believed that the GM shapes the composition and diversity of intestinal microbiota by participating in the breakdown of dietary fiber and the production of short-chain fatty acids (SCFAs)^[25]^. Studies suggest that SCFAs, such as acetate, propionate, and butyrate, derived from GM play a significant role in BP regulation through various mechanisms and are essential in cardiovascular homeostasis. SCFAs interact with some receptors, influencing vasodilation and neural feedback in the gut, ultimately affecting BP levels^[26,27]^. Research indicates that SCFAs can activate different receptors, leading to complex and diverse effects on BP regulation. For instance, SCFAs binding to GPR41 can induce vasodilation and lower BP, while binding to Olfr78 may increase BP levels^[28]^. Moreover, SCFAs have been linked to the expression of genes involved in BP regulation, highlighting their potential as a target for novel prevention and treatment methods for hypertension^[26]^. Besides SCFAs, other microbial metabolites that have been investigated for their effects on BP regulation include trimethylamine-N-oxide (TMAO) and bile acids (BAs). TMAO has been shown to influence BP, with studies demonstrating its BP-lowering effects in patients with resistant hypertension^[29]^. Additionally, gut bacteria and their metabolites may affect BP variability through systemic mechanisms outside the intestine^[8]^. BAs have been implicated in BP regulation. Serum total bile acid (TBA) has been associated with new-onset hypertension during pregnancy, where high TBA levels were positively related to systolic and diastolic BP, and proteinuria^[30]^. Furthermore, some BAs have vasodilatory effects; for instance, taurocholic acid has been shown to induce vasodilation in mesenteric and aortic rings^[31]^.

In general, the human GM plays a vital role in various physiological processes, and research on this topic is ongoing. Recent studies have brought to light the importance of the GM in the etiology and development of hypertension. Dysbiosis of the GM is linked to a reduction in the expression of tight junction proteins, a decrease in the quantity of goblet cells, a breakdown of the gut epithelial barrier, and a decrease in mucus in the intestinal lumen, suggesting that it might contribute to the development of hypertension^[32]^. Hypertension has been correlated with GM dysbiosis, which includes an increased abundance of Gram-negative microbiota^[33]^. Through a variety of mechanisms, the GM can control BP by including host-induced modifications to microbiome-associated gene pathways, such as using G protein-coupled receptors to activate many downstream signaling pathways^[26]^. Both in humans and animals, the relationship between GM and hypertension has been noted, and microbial genera that influence BP have been identified^[34]^.

Animal studies have indicated that the GM plays an important role in the regulation of hypertension. For instance, a study discovered that raised BP was linked to the prevalence of Firmicutes and Bacteroidetes in gut by using several hypertension models, including rats that were spontaneously hypertensive and Dahl salt-sensitive^[35]^. Numerous large-cohort investigations have revealed inconsistent microbiological indicators, and the connection between GM and hypertension still has to be clarified^[36]^. However, some studies have identified specific microbes and metabolic pathways that may help explain the protective effect of probiotics (live microorganisms, typically bacteria or yeast, that confer health benefits) in treating hypertension; in fact, the metagenomic analysis revealed that the increased *Lawsonia* and *Pyrolobus*, and reduced *Alistipes* and *Alloprevotella* levels were tightly correlated with lowered BP, suggesting that these bacteria could affect BP by changing steroid hormone levels^[37]^.

It has been determined that several microbial genera influence BP. Blood pressure is correlated with increases in *Lactobacillus*, *Roseburia*, *Coprococcus*, *Akkermansia*, and *Bifidobacterium*, and inversely correlated with increases in *Streptococcus*, *Blautia*, and *Prevotella*^[34]^. Furthermore, several bacteria can stimulate the synthesis of trimethylamine-N-oxide (TMAO), a metabolite that is currently the subject of extensive research in the field of hypertension^[38]^. Specifically, *Enterobacteriaceae* have been shown to promote the growth of bacteria that produce trimethylamine (TMA) from TMAO, while certain lactic acid bacteria also exhibit increased lactate production when grown in the presence of TMAO^[39]^. Additionally, other bacterial strains like *Anaerococcus hydrogenalis*, *Clostridium asparagiforme*, *Clostridium hathewayi*, and *Clostridium sporogenes* have been linked to TMA generation^[40]^. The GM plays a critical role in TMAO metabolism, and understanding how bacterial communities influence TMAO levels could inform new therapeutic approaches to manage conditions such as hypertension and cardiovascular disease^[41,42]^.

## WHAT IMPACT DOES THE GUT MICROBIOTA HAVE ON BLOOD PRESSURE?

Numerous studies using germ-free mouse models have provided compelling evidence of the causal relationship between the GM and BP regulation^[43,44]^. These investigations have demonstrated that the GM has an essential role in BP control and that altering the microbiota may be a promising treatment for hypertension. Utilizing gnotobiotic, or germ-free, mice has proven crucial in this field. The mice exhibit blunted responses to angiotensin II and experience elevated blood pressure after receiving fecal transplants from hypertension patients instead of normotensive donors. In both genetic and pharmacological models, restoration of normal microbiota reduces and prevents the development of hypertension, and experimental hypertension is typically associated with alterations in the composition of the GM^[45]^. Comparing germ-free rats to their conventionalized counterparts, the lack of microbiota led to relative hypotension, indicating that GM is essential for BP regulation. When microbiota was introduced to germ-free rats, both BP and vascular contractility returned, suggesting that microbiota might influence BP via a vascular-dependent mechanism^[46]^.

It remains to be seen how the GM influences BP. Global knowledge about the gut microbiome’s role in hypertension may offer crucial insights into its prevention. Recently, it was demonstrated that the human breast milk-derived bacteria, administered orally, could lower BP and alter metabolites in a mouse model of elevated BP induced by high fructose, suggesting a potential therapeutic approach^[37]^.

The interplay between the gut microbiome and the immune system is complex and dynamic, and it has significant implications for health and disease, including the regulation of inflammatory responses and the maintenance of immune homeostasis^[47]^. Studies have shown that anti-inflammatory and antioxidant properties can help regulate BP. A disruption in the equilibrium between oxidants and antioxidants is known as oxidative stress, and it has been connected to the emergence of endothelial dysfunction, inflammation, and heightened vascular contractility, which in turn leads to the remodeling of cardiovascular tissue^[48]^. Antioxidants and microbial components, such as carotenoids, polyphenols, vitamins, and sterols, have been shown to restore the proper functioning of vessels and reverse the harmful effects of free radicals. Additionally, flavonoids have been reported to have vasodilatory and antihypertensive effects^[49]^. The phytochemical indole-3-carbinol, which is obtained from cruciferous vegetables, has demonstrated anti-inflammatory, antioxidant, antihypertensive, and antiarrhythmic properties^[50]^. Anti-inflammatory and antioxidant properties of some microbial metabolites may help regulate BP and have great potential for therapeutic treatments^[51]^. For instance, SCFAs like acetate and butyrate have been observed to have anti-inflammatory effects on myeloid and intestinal epithelial cells, potentially benefitting BP regulation^[52]^. Additionally, SCFA-producing bacteria like *Faecalibacterium* and *Coprococcus* have been associated with lower BP and better mental health outcomes^[53]^.

It is interesting to note that some GM plays a crucial role in metabolizing certain nutrients, such as nitrates from vegetables, and converting them into nitric oxide, a vasodilator molecule that helps relax blood vessels and regulate BP. This process involves the microbial reduction of nitrates to nitrites and further to nitric oxide, which has been shown to contribute to the maintenance of cardiovascular health and the regulation of BP^[38]^. The microbially-derived NO can cross the epithelial barriers, reaching the portal vein and eventually entering the systemic circulation^[54]^. The production of nitric oxide from dietary nitrates by the GM represents an important link between diet, the gut microbiome, and cardiovascular function, highlighting the potential impact of microbial metabolism on human health^[8]^.

The gut microbiome can also play a significant role in the metabolism, pharmacokinetics, and efficacy of antihypertensive medications. The GM can influence the pharmacokinetics of antihypertensive drugs, affecting their absorption, distribution, and elimination. This can lead to changes in drug concentrations and may impact the overall effectiveness of the medication^[55]^. Understanding these interactions is crucial for optimizing the treatment of hypertension and addressing the challenges associated with drug resistance. Antihypertensive medications can interact with the gut microbiome, which may affect drug pharmacokinetics and the efficacy of BP-lowering drugs^[56]^. Resistant hypertension is a condition where BP remains high despite taking at least three different antihypertensive drugs at optimal doses. Understanding the interactions between different medications and lifestyle factors is crucial for optimizing the treatment of hypertension and addressing the challenges associated with drug resistance^[57]^.

The GM plays a role in the metabolism of antihypertensive drugs, which can contribute to drug resistance^[58]^. For example, antihypertensive drugs such as amlodipine and nifedipine can be metabolized by gut microbial enzymes, influencing drug absorption, and leading to changes in their pharmacokinetic parameters^[59]^. The GM has been shown to be involved in the development of antihypertensive drug resistance, which can lead to a reduced response to antihypertensive medications^[58]^. Depletion of the GM, achieved through antibiotic treatment, has been shown in a patient to enhance the BP-lowering effect of certain antihypertensive drugs, suggesting that the GM plays a significant role in their efficacy^[60]^.

Antihypertensive drugs such as losartan, valsartan, and telmisartan can be transformed by GM in the *in vitro* setting, suggesting potential interactions with the gut microbiome. However, the specific metabolites generated and their effects on the efficacy of the drugs are not clear and may represent secondary effects due to the complex interplay between antihypertensive agents and GM. Interestingly, the angiotensin receptor antagonist losartan reduced gut dysbiosis and improved gut integrity in an experimental model^[61]^. Some medications may affect gut microbiome composition and gut epithelial barrier, as endothelial dysfunction and attenuation of BP may be increased in response to angiotensin^[62]^. Captopril (angiotensin-converting enzyme inhibitor) reshaped the gut microbiome composition, and following its withdrawal, there was a noticeable alteration in microbial composition, with *Parabacteroides*, *Mucispirillum*, and *Allobaculum* being the most prominently increased genera^[63]^. Additionally, the medication reduced gut inflammation and permeability. The metoprolol (beta-blocker) hypertension treatment might affect BP by changing levels of gut microbiome-derived metabolites in patients with hypertension^[64]^, and the medication enalapril might be involved in BP regulation by changing gut microbiome composition and reducing blood TMAO levels in an experimental model^[65]^.

A recent study has shown how complex and multifaceted the relationship is between BP, antihypertensive drugs, and GM^[55]^. It is expected that a significant percentage (more than 10%) of hypertension patients show resistance to standard treatments^[66]^. The pathophysiological processes and etiology of resistant hypertension are still not well understood. Thus, additional investigation is necessary to have a more profound comprehension of these processes and may reveal possible therapeutic consequences resulting from these intricate relationships.

## PREBIOTIC AND PROBIOTIC INTERVENTIONS

Many studies have highlighted the importance of prebiotics, which are components that selectively promote the proliferation and/or activity of specific bacteria in the gut, improving host health^[67]^. According to recent studies, prebiotics may have a role in preventing and/or lowering hypertension based on experimental findings. Prebiotics have been shown to reduce the incidence of hypertension through a variety of strategies, but one particularly noteworthy one is their impact on blood lipid and cholesterol levels, which are both strongly associated with hypertensive disorders^[68]^. A possible mechanism for the lipid and cholesterol-lowering benefits of prebiotics is the synthesis of SCFAs. This interaction highlights one possible way that prebiotics support the regulation of BP. Prebiotics have been shown to be effective in lowering the risk of hypertension by aiding in the gastrointestinal tract’s ability to absorb minerals such as calcium^[69]^. This comprehensive strategy highlights the potential of prebiotics to affect multiple physiological systems linked to BP management in addition to altering GM. Furthermore, new studies demonstrate how a high-fiber prebiotic supplement can lower BP in people with hypertension due to changes in the makeup of gut bacteria^[68]^.

Probiotics are living microorganisms that provide health advantages to the host when ingested in appropriate amounts^[70]^. Several probiotic bacterial strains are ingested for their health benefits. Examples of such strains include those belonging to the genera *Lactobacillus*, *Bifidobacterium*, *Saccharomyces*, *Enterococcus*, *Streptococcus*, *Pediococcus*, *Leuconostoc*, and *Bacillus*. According to various studies, incorporating probiotics into diet could potentially lead to a reduction in BP, particularly among individuals with initially elevated levels^[71]^. Probiotic supplements dramatically lowered systolic and diastolic BP, according to other studies^[72]^, notably in individuals with hypertension and type 2 diabetes. In addition, it has been discovered that two probiotic strains, *Lactobacillus rhamnosus* and *Bifidobacterium lactis*, which are found in foods like cheese and yogurt, may help decrease BP^[37]^. Utilizing both probiotics and prebiotics together offers a synergistic approach to optimally modify GM and potentially exert greater BP-reducing effects than either alone. These results suggest that prebiotics and probiotics may help lower BP, but more studies are needed to confirm their effectiveness. Remarkably, patients’ prebiotic and probiotic choices should be customized according to their unique circumstances, including age, sex, ethnicity, food preferences, way of life, and amount of physical activity they engage in each day^[73,74]^.

## CONCLUSION

The microbiota plays a fundamental role in controlling hypertension, exerting a direct influence on various aspects of the cardiovascular system and health. In addition to traditional factors such as diet and lifestyle, the microbial community in the gastrointestinal tract has a notable impact on BP regulation. Recent studies have revealed the complex interaction between the microbiota and BP control. Beneficial bacteria in the gut produce metabolites, such as SCFAs, that play a crucial role in hypertension control. These compounds have anti-inflammatory and antioxidant properties, helping regulate BP. The GM also plays a role in metabolizing certain nutrients, such as nitrates from vegetables, converting them into nitric oxide, a vasodilator molecule that helps relax blood vessels and regulate BP. Furthermore, the GM influences the immune system by modulating systemic inflammatory responses. Chronic inflammation is associated with the development of hypertension, and the microbiota plays a key role in regulating this inflammatory state. Therefore, understanding the importance of the microbiota in hypertension control offers an innovative perspective on the therapeutic and preventive approach to this condition. Targeted strategies to promote a healthy microbiota, through dietary interventions, probiotics, and prebiotics, can become valuable tools in managing BP and promoting health in a holistic manner.

## DECLARATIONS

### Copyright

© The Author(s) 2024.
